# Processes in Pathology and Microbiology

**Published:** 1984-07

**Authors:** J. D. Davies

**Affiliations:** Department of Pathology University of Bristol


### Book Reviews

**PROCESSES IN PATHOLOGY AND MICROBIOLOGY**
**M. J. Taussig**
**Blackwell Scientific Publications, Oxford, 2nd Edition, 1984**
**pp. 914 + xi, hardback. Price £15.00**

Nothing stays the same. Even the most conservative must agree with McLuhan's message that the medium itself can change the original.[1] Do you remember the awful texts that Messrs. J. & A. Churchill used to produce? A sort of laborious copper plate transmuted into post-war printers' copy. 'Recent Advances in Whatever' in the late 1950's must have looked, even at that time, as if they were more the 'Last Steps to the Grave'.

This book on 'Processes in Pathology and Microbiology' is not presented in that funereal manner. The publishers have used modern typeface, good usage of tables in the layout, diagrams and even a generous three-quarter page of text to enliven the appearance of the book. A plasticised hard-back cover completes the package. £15 for 914 pages can't be counted as expensive today.

Most Book Reviews, at least in medical journals, start off the other way round. Content first; presentation second. I am purposely doing this one the other way round.

How about the content? It so happens that recently the Bristol Medical School Library has had a Bumper Free Offer of old spare copies to pick up, if you want, on leaving the premises. Few bibliophiles could possibly resist the temptations. I was especially interested to see Valy Menkin's classic on Inflammation (c. 1950). It brought back the ambience of General Pathology texts of that era. Hence, to be truthful, this review.

What has happened since? The front runners have probably been (at least in this country) Boyd,[2] Payling Wright,[3] Florey,[4] Walter and Israel,[5] and the current American favourite from Robbins.[6]

Is there any way of classifying the trend? How about the following subjective assessment in pseudo-scientific tabular form as shown below.

We apparently live in an age of increasing didacticism and the need for acceptable accuracy. Traditional academic discourse and encyclopaedic coverage don't seem, either to publishers or buyers, to be important. Everyone loves their own eccentric, but not everyone anyone else's. Orthodoxy rules.

What clues are there from the titles in these three decades? Their Key Words are:

| | *Key Word in title* | | *Comment* |
|---|---|---|---|
| A | Textbook[2] | ....... | reassuring |
| B | Introduction[3] | ....... | modest |
| C | General[4] | ....... | impressive |
| D | General[5] | ....... | ditto |
| E | Basis | ....... | basic |
| F | Processes | ....... | echoes of computing and essentially ongoing in the modern idiom. |

By now you will have got the message. This worthy text by Taussig is a tool for the times. Our own preclinical medical students have for several years admitted that more of them have spent Grant Money on the first edition of this textbook than on any other in the field. Postgraduate pathology trainees evidently feel much the same.

Should anyone want an update in the way general pathology is now taught, or potentially applied, this is the best of the current field. The factual content and biomedical background are not easy going. With

**Scores out of 10**

| | | *Complete coverage* | *Eccentricity of view* | *Obvious error in account* | *Contrapuntal argument* | *Didactic style* |
|---|---|---|---|---|---|---|
| A | Boyd[2] | 8 | 27 | 4 | 5 | 5 |
| B | Payling Wright[3] | 10 | 4 | 1 | 10 | 6 |
| C | Florey[4] | 10 | 5 | 2 | 10 | 5 |
| D | Walter & Israel[5] | 10 | 0 | 1 | 2 | 8 |
| E | Robbins[6] | 10 | 0 | 0 | 1 | 9 |
| F | Taussig | 7 | 4 | 0 | 0 | 10 |

the help of Albert's 'Molecular Biology of the Cell'[7] you, like your reviewer, will be well equipped to assimilate and even enjoy this text, which is so favoured by our student successors still at Medical School. Clearly this book is an indicator of a current trend in Preclinical Teaching Methods. Few clinicians can afford to ignore the way in which medical students are being instructed before they reach the wards. Why not be bold, and try the book? It could help to bridge the generation gap. You might think the message justifies the medium. Or, maybe the medium makes the message. See for yourself.

J. D. Davies
Department of Pathology
University of Bristol

### REFERENCES

1. MILLER, J. (1971) McLuhan. Fontana/Collins, London.
2. BOYD, W. (1953) A Textbook of Pathology. 6th Ed. H. Kimpton, London.
3. WRIGHT, G. P. (1954) An Introduction to Pathology. 2nd Ed. Longmans Green, London.
4. FLOREY, H. W. (1970) General Pathology. 4th Ed. Lloyd-Luke, London.
5. WALTER, J. B. and ISRAEL, M. S. (1974) General Pathology. 4th Ed. Churchill-Livingstone, Edinburgh.
6. ROBBINS, S. L. (1974) Pathologic Basis of Disease. W. B. Saunders, Philadelphia,.
7. Book Review (1983) *Bristol Med.-Chi. J.* **98**, 201.

